# Interleukin-5 Potentiates Sulfidopeptide Leukotriene Production by Human Eosinophils

**DOI:** 10.1155/S0962935194000098

**Published:** 1994

**Authors:** A. J. M. Van Oosterhout, A. Van Der Poel, L. Koenderman, D. Roos, F. P. Nijkamp

**Affiliations:** 1 Department of Pharmacology Faculty of Pharmacy Utrecht University P.O. Box 80.082 Utrecht 3508 TB The Netherlands; 2 Central Laboratory of the Netherlands Red Cross Blood Transfusion Service and Laboratory for Experimental and Clinical Immunology University of Amsterdam The Netherlands; 3 Department of Pulmonary Disease State University Hospital Utrecht Utrecht The Netherlands

## Abstract

Interleukin-5 (IL-5) has been shown to be a selective eosinophil growth and differentiation factor. In the present study, the effect of recombinant human IL-5 on human eosinophil sulfidopeptide leukotriene production was investigated. IL-5 did not affect leukotriene synthesis in unstimulated eosinophils. However, IL-5 potentiated leukotriene synthesis by eosinophils stimulated with serum treated zymosan (STZ) or the calcium ionophore A23187 by 69% and 135%, respectively. The priming effect of IL-5 was dose dependent, with significant stimulation occurring at 1 000 U/ml for STZ and 100-1 000 U/ml for A23187. Pre-incubation with IL-5 did not increase leukotriene synthesis further.


Mediators of Inflammation 3, 53–55 (1994)

### Interleukin-5 potentiates sulfidopeptide leukotriene production by human eosinophils

A. J. M. Van Oosterhout,[1,CA] A. Van Der Poel,[1] L. Koenderman,[2,*] D. Roos[2] and F. P. Nijkamp[1]

[1]Department of Pharmacology, Faculty of Pharmacy, Utrecht University, P.O. Box 80.082, 3508 TB, Utrecht, The Netherlands; [2]Central Laboratory of the Netherlands Red Cross, Blood Transfusion Service and Laboratory for Experimental and Clinical Immunology, University of Amsterdam, The Netherlands
**Present address*: Department of Pulmonary Disease, State University Hospital Utrecht, Utrecht, The Netherlands

[CA] Corresponding Author

**INTERLEUKIN-5 (IL-5) has been shown to be a selective eosinophil growth and differentiation factor. In the present study, the effect of recombinant human IL-5 on human eosinophil sulfidopeptide leukotriene production was investigated. IL-5 did not affect leukotriene synthesis in unstimulated eosinophils. However, IL-5 potentiated leukotriene synthesis by eosinophils stimulated with serum treated zymosan (STZ) or the calcium ionophore A23187 by 69% and 135%, respectively. The priming effect of IL-5 was dose dependent, with significant stimulation occurring at 1 000 U/ml for STZ and 100–1 000 U/ml for A23187. Pre-incubation with IL-5 did not increase leukotriene synthesis further.**

**Key words:** Eosinophil, Interleukin-5, Leukotriene

### Introduction

Human eosinophils play an important role in the pathogenesis of bronchial asthma.[1,2] Peripheral blood and sputum eosinophilia often accompanies asthma, a large number of eosinophils infiltrate the airways during the late asthmatic reaction and mediators released by these cells can affect airway function.[2] Bronchospasmogenic substances such as sulfidopeptide leukotrienes (LT) and platelet-activating factor (PAF), as well as other eosinophil derived mediators appear to play a role in the development of airway hyperreactivity, a main characteristic of bronchial asthma.[1–3] Eosinophil cytotoxic cationic proteins can damage airway epithelial cells, which may cause airway hyperreactivity.[4–7] Furthermore, inhaled sulfidopeptide leukotrienes and PAF are able to induce airway hyperreactivity in laboratory animals and humans.[8–11]

It has been demonstrated that eosinophils from asthmatic patients are hypodense and produce more leukotriene $C_4$ and reactive oxygen metabolites than those from healthy persons.[2] The mechanism causing the eosinophilia and the primed state of eosinophils in asthmatics is unknown. It has been suggested that T-lymphocyte derived cytokines may play an important role in this phenomenon.[12] T-lymphocytes are activated in acute asthma and infiltrate the airways after allergen provocation,[12] thus creating an ideal environment for eosinophil proliferation and activation.

Several cytokines have been shown to induce eosinophil proliferation, chemotaxis, activation and/or priming.[13–15] Granulocyte macrophage colony-stimulating factor (GM–CSF), tumour necrosis factor (TNF), interleukin-3 (IL-3) and IL-5 have all been shown to regulate one or more eosinophil functions.[14,15] Of these cytokines, only IL-5 seems to be a selective activator of eosinophils, whereas other cytokines also influence neutrophils. It has been shown that IL-5 induces eosinophil proliferation, chemiluminescence, release of cytotoxic cationic proteins, chemotaxis and cytotoxicity, and enhances adhesion to endothelial cells.[13,16–18] In the present study, the influence of IL-5 on serum-treated zymosan (STZ) and calcium ionophore (A23187) induced sulfidopeptide leukotriene production by human eosinophils was investigated.

### Materials and Methods

Eosinophils were isolated from blood of normal human volunteers (Central Laboratory of the Netherlands Red Cross Blood Transfusion Service, Amsterdam and Bloodbank, Utrecht, the Netherlands) as described before in detail.[19] The purity of the eosinophils was 95 ± 3% and the viability was always more than 95% as determined by trypan blue

© 1994 Rapid Communications of Oxford Ltd exclusion. For the production of leukotriene, 200 $\mu$l of eosinophils ($3 \times 10^6$ cells/ml) were incubated (37°C, constant agitation) with or without different concentrations of recombinant human IL-5 (generously provided by Dr C. J. Sanderson, National Institute for Medical Research, Mill Hill, London NW7, UK). The cells were stimulated with either serum treated zymosan (0.5 mg/ml final concentration) for 30 min or with the calcium ionophore A23187 (2.5 $\mu$M) for 10 min in a final volume of 250 $\mu$l. Incubations were terminated by cooling on ice, and supernatants were collected by centrifugation at $8000 \times g$ for 1 min followed by storage under nitrogen at −80°C until analysis. The amount of leukotriene $C_4/D_4/E_4$ in the supernatants was determined with a radioimmunoassay kit according to the manufacturer's instructions (Amersham, Buckinghamshire, UK).

### Results

Incubation of human eosinophils with IL-5 for 30 min at 10–1 000 U/ml itself induced a small but not significant increase in leukotriene synthesis (Table 1). However, IL-5 (1 000 U/ml) potentiated STZ induced leukotriene synthesis by 69% compared with the production of leukotriene in the absence of the cytokine (Fig. 1). IL-5 (300 U/ml) induced a potentiation of the calcium ionophore induced leukotriene synthesis by 135% compared with the production of leukotriene in the absence of the cytokine. Pre-incubation of eosinophils with IL-5 (1 000 U/ml) for 10 min and subsequent stimulation with STZ or A23187 showed a similar potentiation of leukotriene synthesis as measured without pre-incubation (Table 2). In pilot experiments, longer pre-incubation times of eosinophils with IL-5 (up to 60 min) did not show a further enhancement of leukotriene synthesis. Leukotriene synthesis by eosinophils decreased after pre-incubation above 15 min. This was observed both in the absence or presence of IL-5. There were large donor–to–donor variations in eosinophil-leukotriene production and its IL-5 enhancement

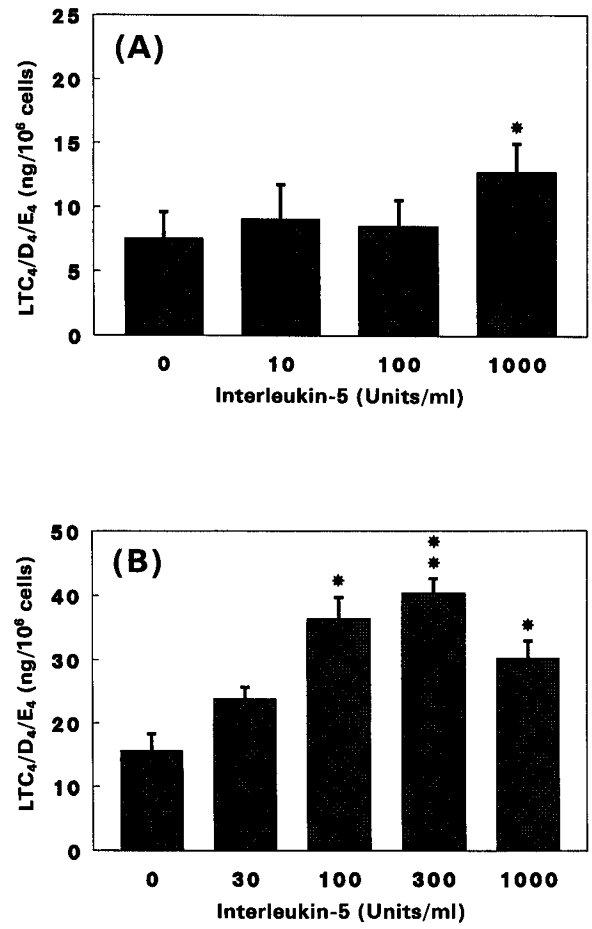

FIG. 1. The effect of different concentrations of IL-5 on human eosinophil leukotriene $C_4/D_4/E_4$ synthesis induced with (A) STZ ($n = 11$) or (B) calcium ionophore A23187 ($n = 4$). Results are presented as means ± S.E.M. IL-5 and the stimulating agents were added simultaneously. $^*p < 0.05$ and $^{**}p < 0.01$ as determined with the paired Student's $t$-test and compared with the leukotriene synthesis in the absence of IL-5.

**Table 1.** The direct effect of different concentrations of IL-5 on human eosinophil leukotriene $C_4/D_4/E_4$ synthesis ($n = 3$)

| IL-5 (U/ml) | LT synthesis (pg/$10^6$ cells) |
|---|---|
| 0 | 36.7 ± 3.6 |
| 10 | 37.5 ± 3.2 |
| 100 | 42.3 ± 4.0 |
| 1 000 | 47.9 ± 2.7 |

Results are presented as means ± S.E.M.

**Table 2.** The effect of pre-incubation time on human eosinophils with IL-5 on STZ- or A23187-induced leukotriene $C_4/D_4/E_4$ synthesis

| Pre-incubation time (min) | STZ | | A23187 | |
|---|---|---|---|---|
| | −IL-5 | +IL-5 | −IL-5 | +IL-5 |
| 0 | 8.6 ± 4.0 | 13.1 ± 5.7* | 15.8 ± 6.4 | 56.5 ± 14.8* |
| 10 | 9.2 ± 4.2 | 14.5 ± 5.2** | 12.4 ± 4.2 | 52.9 ± 6.8* |

$^*p < 0.05$ and $^{**}p < 0.01$ as determined with the paired Student's $t$-test and compared with the control. Results (ng/$10^6$ cells) are expressed as mean ± S.E.M. ($n = 6$ for STZ, $n = 3$ for A23187). IL-5 was used as 1 000 U/ml.

**Table 3.** Three individual experiments illustrating the donor-to-donor variation in STZ-induced leukotriene $C_4/D_4/E_4$ synthesis (ng/$10^6$ cells) and the enhancement by IL-5 in human eosinophils. IL-5 and the stimulating agents were added simultaneously

| IL-5 (U/ml) | Leukotriene concentration (ng/$10^6$ cells) | | |
|---|---|---|---|
| | Donor 1 | Donor 2 | Donor 3 |
| 0 | 1.38 | 6.32 | 3.55 |
| 10 | 3.09 | 16.83 | 2.00 |
| 100 | 2.69 | 12.26 | 2.24 |
| 1 000 | 5.37 | 20.55 | 8.70 |

(Table 3). The reason for this variability is unclear at present, but others have reported similar donor variation.[15]

### Discussion

In the present study, it is demonstrated that IL-5 potentiates leukotriene production of human eosinophils stimulated with serum treated zymosan or calcium ionophore. It has been shown that GM–CSF enhances A23187-induced leukotriene synthesis by eosinophils by approximately 135%.[15] However, in contrast to the priming of eosinophil leukotriene production by GM–CSF,[15] the priming by IL-5 does not increase with time. Similar immediate priming effects have been demonstrated in the basophil with IL-5 induced potentiation of histamine release and leukotriene generation.[20,21]

The potentiation of A23187 induced leukotriene synthesis by IL-5 is more evident and occurs at lower concentrations than STZ induced leukotriene synthesis. The reason for this difference is unclear but could be due to differences in the signal transduction pathways between STZ and A23187 stimulation.

It has been demonstrated that eosinophils from asthmatic patients have an increased capacity to produce leukotriene after stimulation.[2] Other eosinophil functions in asthmatics such as the production of reactive oxygen metabolites appear to be primed as well.[2,12] T-lymphocyte derived cytokines are potential candidates for the induction of a primed state in eosinophils in asthma as these cells are activated in acute asthma.[12] IL-5 mRNA expression has been demonstrated in the bronchial mucosa and bronchoalveolar lavage cells of asthmatics.[22,23] Eosinophil derived mediators seem to play an important role in the development of airway hyperreactivity.[1–3] Interestingly, we demonstrated that antibody to IL-5 prevented the antigen-induced airway hyperreactivity and eosinophil infiltration in ovalbumin sensitized guinea-pigs.[24] Vice versa, administration of IL-5 to guinea-pigs induced airway eosinophilia and hyperreactivity.[24,25] Based on these data, together with the present findings, it can be speculated that IL-5 may play an important role in the pathogenesis of asthma.

### References

1. Gleich GJ. The eosinophil and bronchial asthma: current understanding. *J Allergy Clin Immunol* 1990; **85**: 422–436.
2. Djukanovic R, Roche WR, Wilson JW, Beasley CRW, Twentyman OP, Howarth PH, Holgate ST. Mucosal inflammation in asthma. *Am Rev Respir Dis* 1990; **142**: 434–457.
3. Van Oosterhout AJM, Nijkamp FP. Minireview: lymphocytes and bronchial hyperresponsiveness. *Life Sci* 1990; **46**: 1255–1264.
4. Frigas E, Loegering DA, Gleich GJ. Cytotoxic effects of the guinea pig eosinophil major basic protein on tracheal epithelium. *Lab Invest* 1980; **42**: 35–43.
5. Motojima S, Frigas E, Loegering DA, Gleich GJ. Toxicity of eosinophil cationic proteins for guinea pig tracheal epithelium *in vitro*. *Am Rev Respir Dis* 1989; **139**: 801–805.
6. Gundel RH, Letts LG, Gleich GJ. Human eosinophil major basic protein induces airway constriction and airway hyperresponsiveness in primates. *J Clin Invest* 1991; **87**: 1470–1473.
7. Uchida DA, Ackrman SJ, Coyle AJ, Larsen GL, Weller PF, Freed J, Irvin CG. The effect of human eosinophil granule major basic protein on airway responsiveness in the rat *in vivo*. *Am Rev Respir Dis* 1993; **147**: 982–988.
8. Arm JP, Spur BW, Lee TH. The effects of inhaled leukotriene $E_4$ on the airway responsiveness to histamine in subjects with asthma and normal subjects. *J Allergy Clin Immunol* 1988; **82**: 654–660.
9. O'Hickey SP, Hawksworth RJ, Fong CY, Arm JP, Spur BW, Lee TH. Leukotrienes $C_4$, $D_4$, and $E_4$ enhance histamine responsiveness in asthmatic airways. *Am Rev Respir Dis* 1991; **144**: 1053–1057.
10. Jacques CAJ, Spur BW, Johnson M, Lee TH. The mechanism of $LTE_4$-induced histamine hyperresponsiveness in guinea-pig tracheal and human bronchial smooth muscle, *in vitro*. *Br J Pharmacol* 1991; **104**: 859–866.
11. Patterson R, Harris KE, Bernstein PR, Krell RD. Aerosolized leukotriene $D_4$ converts monkeys that are negative aerosolized *Ascaris* responders to positive airway responders. *Life Sci* 1986; **38**: 1179–1184.
12. Frew AJ, Kay AB. Eosinophils and T-lymphocytes in late-phase allergic reactions. *J Allergy Clin Immunol* 1990; **85**: 533–539.
13. Lopez AF, Sanderson CJ, Gamble JR, Campbell HR, Young IG, Vadas MA. Recombinant human interleukin-5 is a selective activator of human eosinophil function. *J Exp Med* 1988; **167**: 219–224.
14. Silberstein DS, David JR. The regulation of human eosinophil function by cytokines. *Immunol Today* 1987; **8**: 380–385.
15. Silberstein DS, Owen WF, Gasson JC *et al.* Enhancement of human eosinophil cytotoxicity and leukotriene synthesis by biosynthetic (recombinant) granulocyte-macrophage colony-stimulating factor. *J Immunol* 1986; **137**: 3290–3294.
16. Fujisawa T, Abu-Ghazaleh R, Kita H, Sanderson CJ, Gleich GJ. Regulatory effect of cytokines on eosinophil degranulation. *J Immunol* 1990; **144**: 642–646.
17. Walsh GM, Hartnell A, Wardlaw AJ, Kurihara K, Sanderson CJ, Kay AB. IL-5 enhances the *in vitro* adhesion of human eosinophils, but not neutrophils, in a leucocyte integrin (CD11/18)-dependent manner. *Immunology* 1990; **71**: 258–265.
18. Wang JM, Rambaldi A, Biondi A, Chen ZG, Sanderson CJ, Mantovani A. Recombinant human interleukin-5 is a selective eosinophil chemoattractant. *Eur J Immunol* 1989; **19**: 701–705.
19. Koenderman L, Kok PTM, Hamelink ML, Verhoeven AJ, Bruijnzeel PLB. An improved method for the isolation of eosinophilic granulocytes from peripheral blood of normal individuals. *J Leukocyte Biol* 1988; **44**: 79–86.
20. Bischoff SC, Brunner T, Deweck AL, Dahinden CA. Interleukin-5 modifies histamine release and leukotriene generation by human basophils in response to diverse agonists. *J Exp Med* 1990; **172**: 1577–1582.
21. Hirai K, Yamaguchi M, Misaki Y, *et al.* Enhancement of human basophil histamine release by interleukin-5. *J Exp Med* 1990; **172**: 1525–1528.
22. Hamid Q, Azzawi M, Ying S, *et al.* Expression of messenger RNA for interleukin-5 in mucosal bronchial biopsies from asthma. *J Clin Invest* 1991; **87**: 1541–1546.
23. Robinson DS, Hamid Q, Ying S, *et al.* Predominant Th2-like bronchoalveolar T-lymphocyte population in atopic asthma. *New Engl J Med* 1992; **326**: 298–304.
24. Van Oosterhout AJM, Ladenius ARC, Savelkoul HFJ, Van Ark I, Delsman KC, Nijkamp FP. Effect of anti-IL-5 and IL-5 on airway hyperreactivity and eosinophils in guinea pigs. *Am Rev Respir Dis* 1993; **147**: 548–552.
25. Van Oosterhout AJM, Van Ark I, Hofman G, Savelkoul HFJ, Nijkamp FP. Recombinant interleukin-5 induces *in vivo* airway hyperresponsiveness to histamine in guinea pigs. *Eur J Pharmacol* 1993; **236**: 379–383.

ACKNOWLEDGEMENT. This study was supported by a research grant of the Dutch Asthma Foundation (87.29). The authors thank R. Ladenius for carefully reading this manuscript.

**Received 2 September 1993; accepted in revised form 24 November 1993**
